# Genomic and Phylogenetic Analysis of *Lactiplantibacillus plantarum* L125, and Evaluation of Its Anti-Proliferative and Cytotoxic Activity in Cancer Cells

**DOI:** 10.3390/biomedicines9111718

**Published:** 2021-11-19

**Authors:** Konstantinos Tegopoulos, Odysseas Sotirios Stergiou, Despoina Eugenia Kiousi, Margaritis Tsifintaris, Ellie Koletsou, Aristotelis C. Papageorgiou, Anthoula A. Argyri, Nikos Chorianopoulos, Alex Galanis, Petros Kolovos

**Affiliations:** 1 Department of Molecular Biology and Genetics, Faculty of Health Sciences, Democritus University of Thrace, 68100 Alexandroupolis, Greece; konstego1@mbg.duth.gr (K.T.); odysster1@mbg.duth.gr (O.S.S.); dkiousi@mbg.duth.gr (D.E.K.); mtsifintaris@gmail.com (M.T.); ellie060496@gmail.com (E.K.); apapage@mbg.duth.gr (A.C.P.); 2 Institute of Technology of Agricultural Products, Hellenic Agricultural Organization DIMITRA, Sofokli Venizelou 1, Lycovrissi, 14123 Attiki, Greece; anthi.argyri@gmail.com (A.A.A.); nchorianopoulos@gmail.com (N.C.)

**Keywords:** *Lactiplantibacillus plantarum*, genomics, whole-genome sequencing, probiotics, comparative genomics, phylogenetic analysis, anti-proliferative activity

## Abstract

*Lactiplantibacillus plantarum* is a diverse species that includes nomadic strains isolated from a variety of environmental niches. Several *L. plantarum* strains are being incorporated in fermented foodstuffs as starter cultures, while some of them have also been characterized as probiotics. In this study, we present the draft genome sequence of *L. plantarum* L125, a potential probiotic strain presenting biotechnological interest, originally isolated from a traditional fermented meat product. Phylogenetic and comparative genomic analysis with other potential probiotic *L. plantarum* strains were performed to determine its evolutionary relationships. Furthermore, we located genes involved in the probiotic phenotype by whole genome annotation. Indeed, genes coding for proteins mediating host–microbe interactions and bile salt, heat and cold stress tolerance were identified. Concerning the potential health-promoting attributes of the novel strain, we determined that *L. plantarum* L125 carries an incomplete plantaricin gene cluster, in agreement with previous in vitro findings, where no bacteriocin-like activity was detected. Moreover, we showed that cell-free culture supernatant (CFCS) of *L. plantarum* L125 exerts anti-proliferative, anti-clonogenic and anti-migration activity against the human colon adenocarcinoma cell line, HT-29. Conclusively, *L. plantarum* L125 presents desirable probiotic traits. Future studies will elucidate further its biological and health-related properties.

## 1. Introduction

*Lactiplantibacillus plantarum* is one of the 26 phylogenetic groups of the *Lactobacillaceae* family that consists of facultative anaerobic, Gram-positive, non-motile and non-spore-forming rods that can occur single, in pairs or short chains, presenting high genomic diversity [1]. The *L. plantarum* group forms a monophyletic clade with other heterofermentative *Lactobacillus* and *Pediococcus* strains and also shares major metabolic attributes with homofermentative lactobacilli [2]. Two subspecies of this species have been identified so far: *L. plantarum* subsp. *plantarum* and *L. plantarum* subsp. *argentoratensis* [2]. *L. plantarum* strains generally present a nomadic lifestyle, as they can be found free living in nutrient-rich environments, such as vegetables or in association with vertebrate or invertebrate hosts [3].

In association with the host, *L. plantarum* strains have been found to attach to and transiently colonize the gut. Several strains can adhere directly onto the intestinal epithelium or mucins, using adhesins and adhesin-like molecules of their cellular surface, in chicken [4], murine [5] and human gut [6]. Persistence in the gastrointestinal (GI) tract is a prerequisite for these interactions to occur, and thus several in vitro and in vivo studies investigated the stress tolerance of novel strains [7]. In this context, genes coding for proteins that mediate bile salt and bile acid resistance have been located previously in the genome of *L. plantarum* strains [8], as well as several proton pumps mediating tolerance to the extremely acidic pH of the stomach [9].

Strains that can survive gastrointestinal transit and colonize the mucosa of the host are further examined for their potential health-promoting benefits after ingestion in a series of in vitro, in vivo and clinical tests. The microorganisms that possess these attributes can then be termed probiotics [10]. One of the most studied aspects of the probiotic character is the ability of strains to inhibit pathogen colonization and expansion. In this vein, symbiotic gut bacteria, including probiotics, can exclude pathogen attachment and colonization by occupying important adhesion spots at the mucosa or intestinal epithelium [11]. Furthermore, probiotic strains can produce a variety of bioactive molecules with antimicrobial action, such as bacteriocins. Indeed, several *L. plantarum* strains are found to possess clusters for bacteriocin synthesis that can limit the proliferation of food spoiling and/or clinically relevant bacteria [12]. Another attribute of potentially probiotic strains is the ability to inhibit the proliferation of cancer cell lines [13] or to induce anti-tumor effects in animal models [14]. Overall, these effects are strain- and cancer cell type-specific and are usually mediated by cell surface molecules or excreted signaling molecules [15].

Strains that present potential probiotic attributes are of great interest to the functional food industry. Indeed, several *L. plantarum* strains have been employed as starters or adjunct starter cultures of dairy [16] and non-dairy [17] fermented foodstuffs. In this context, it is of the utmost importance to ensure that a novel strain can withstand the manufacturing process and storage conditions prior to application in the food industry. For that reason, in silico analysis can support in vitro and in situ experiments by the identification of gene clusters coding for heat and cold stress tolerance. Indeed, several studies have located these clusters in the genome of *L. plantarum* strains intended for biotechnological applications [18].

The high accessibility of sequencing platforms has tremendously accelerated the discovery of novel strains with industrial and/or biotechnological interest, as probiotic phenotypes can be traced back to specific genes and genetic clusters. Here, we present the whole genome sequence of *L. plantarum* L125, a novel potential probiotic strain isolated from a traditional fermented sausage [19]. *L. plantarum* L125 has exhibited favorable probiotic traits, including tolerance to low pH, bile salts and partial bile salt hydrolase activity [19], and was successfully incorporated in dry-fermented pork sausages as an adjunct starter culture [20]. In this study, we describe the phylogenetic relationships of the novel strain and characterize genetic clusters involved in host–microbe interactions, stress tolerance and bacteriocin production. Furthermore, we describe the ability of cell-free culture supernatants (CFCS) of *L. plantarum* L125 to inhibit the proliferation and migration of the human colon adenocarcinoma cell line, HT-29, and investigate the presence of genes potentially involved in this phenotype, thus unveiling its potential health impact.

## 2. Materials and Methods

### 2.1. Bacterial Strain, Culture Conditions and DNA Isolation

*L. plantarum* L125 was isolated from a traditional fermented meat product [19] and was provided by the Institute of Technology of Agricultural Products, Hellenic Agricultural Organization DIMITRA (Likovrisi, Attiki, Greece). *L. rhamnosus* GG ATCC 53103 was acquired from DSMZ (Braunschweig, Germany). Both *Lactobacillus* strains were incubated in de Man, Rogosa, and Sharpe (MRS) broth (Condalab, Madrid, Spain) at 37 °C under anaerobic conditions, prior to DNA extraction. For DNA extraction, *L. plantarum* L125 cells were harvested by centrifugation at 8000× *g* for 4 min. Total genomic DNA was extracted from the pellets using the NucleoSpin^®^ Tissue kit (Macherey-Nagel, Düren, Germany), according to the manufacturer’s instructions. The purity and quantity of the isolated DNA were determined spectrophotometrically at 260 nm using NanoDrop^®^ ND-1000 UV-Vis Spectrophotometer (Thermo Fisher Scientific, Waltham, MA, USA).

### 2.2. Whole-Genome Sequencing and Genome Annotation

The genomic DNA of *L. plantarum* L125 was sequenced using the Illumina NovaSeq6000 (2 × 151 paired ends) platform. The sequencing process resulted in 9,117,708 paired-end reads. FASTQC (version 0.11.9) was used for the estimation of the quality of the reads [21], while reads that did not meet quality criteria were discarded using Trimmomatic (version 0.39) [22]. The de novo assembly procedure was carried out using SPAdes (version 3.15.1) [23], choosing the “--careful” option to minimize the number of mismatches. SSPACE_Standard (version 3.0) was utilized for scaffolding contigs along with the parameter to keep contigs with a minimum length of 500 base pairs [24].

*L. plantarum* L125 genome was annotated locally by Prokaryotic Genome Annotation Pipeline (PGAP) using default parameters [25]. Functional classification of proteins into Clusters of Orthologous Groups (COGs) was executed with the EggNOG-mapper tool (version 2.0), available online at the EggNOG database (version 5.0) [26]. Kyoto Encyclopedia of Genes and Genomes (KEGG) Orthology (KO) assignment of the predicted genes was performed by BlastKOALA (version 2.2) [27]. Pathways of interest were reconstructed by the “Reconstruct” KEGG mapping tool (version 5) [28]. The CAZy database [29] was scanned to detect carbohydrate-active enzymes (CAZymes).

CRISPRDetect (version 2.4) was utilized for the detection of Clustered regularly interspaced palindromic repeats (CRISPR) within the bacterial assembly [30]. PHAge Search Tool Enhanced Release (PHASTER) was used to identify and annotate putative prophage sequences [31]. The Artemis tool (version 18.1.0) [32] was employed to visualize the genome assembly, while its metrics were calculated with the Quality Assessment Tool (QUAST) (version 5.2.0) [33].

### 2.3. Phylogenetic Analysis

Python module Pyani (version 0.2.10) [34] was used to calculate the Average Nucleotide Identity (ANI) between *L. plantarum* L125 and 21 potential probiotic *L. plantarum* strains. The probiotic attributes of the 21 strains are presented in Table S1. MEGAX (version 10.1.8) was used for the phylogenomic analysis, which includes 1000 bootstrap replicates (Maximum Composite Likelihood model) [35]. Neighbor-joining phylogenetic trees were constructed using the online EMBL tool “Interactive Tree of Life” (iTol) (version 6.1.1) [36].

### 2.4. Detection of Genetic Elements Associated with Probiotic Characteristics

BAGEL (version 4) was used to detect and visualize gene clusters that are implicated in the biosynthesis of antimicrobial peptides [37]. The Resistance Gene Identifier (RGI) (version 5.1.1) verified the presence of antibiotic resistance genes [38]. BLAST (basic local alignment search tool) was employed to search for genetic loci that are involved in stress response and host–microbe interactions.

### 2.5. Cell-Free Supernatant Preparation

For the preparation of CFCS, *L. plantarum* L125 was cultured for 20 h in MRS broth at 37 °C under anaerobic conditions. The next day, 10^8^ Colony Forming Units/mL (CFU/mL) were added in Roswell Park Memorial Institute (RPMI)-1640 cell culture medium supplemented with GlutaMAX™, 10% fetal bovine serum (FBS) and 20 mM 4-(2-hydroxyethyl)-1-piperazineethanesulfonic acid (HEPES) (all from Thermo Fisher Scientific, Waltham, MA, USA) and were incubated anaerobically at 37 °C for 24 h. Then, the bacterial cells were pelleted by centrifugation at 2.600× *g* for 15 min, and the supernatants were sterile filtered using an 0.22 μm pore size filter (Corning, New York, NY, USA). The dilution of the CFCS was performed in the cell culture medium without antibiotics.

### 2.6. Sulforhodamine B Colorimetric Assay

The sulforhodamine B (SRB) colorimetric assay was employed to investigate the anti-proliferative potential of CFCS of *L. plantarum* L125, against the human colon adenocarcinoma cell line, HT-29 (ATCC, Manassas, VA, USA). HT-29 cells were maintained in RPMI-1640 medium supplemented with GlutaMAXTM, 10% FBS, 100 μg/mL streptomycin and 100 U/mL penicillin (all from Thermo Fisher Scientific) in a humidified, sterile atmosphere at 37 °C, 5% CO_2_. Cells were seeded in 96-well plates (Corning) at a density of 7000 cells per well. The next day, cells were treated with 100 μL of CFCS (undiluted or diluted to a ratio of 1:2). Untreated cells (control) were maintained in standard cell culture medium. After 24 or 48 h treatments, the SRB assay was performed as previously described [39]. For the calculation of the cellular survival, the following formula: ((sample _OD570_ − media blank _OD570_)/(mean control _OD570_ − media blank _OD570_)) × 100 was applied. The assay was performed four independent times in octuplicates.

### 2.7. Colony Formation Assay

A colony formation assay was performed to determine the anti-clonogenic effect of CFCS on HT-29 cells, as previously described, with minor modifications [40]. Briefly, HT-29 cells (1000 cells per 100 mm plate) were treated with undiluted CFCS from *L. plantarum* L125 or *L. rhamnosus* GG for 48 h. The cells were incubated for 10 days until the formation of visible colonies. The colonies were stained with 0.5% (*v*/*v*) crystal violet, following the protocol proposed by Franken et al. 2006 [41]. Results are expressed as: Number of colonies (%) = (number of colonies _treated_/number of colonies _untreated_) × 100.

### 2.8. Wound Healing Assay

The anti-migration potential of *L. plantarum* L125 CFCS was examined using the wound healing assay. To this end, HT-29 cells were seeded in polymer coverslip inserts in 35 mm µ-Dishes at a density of 80,000 cells per silicone insert (Ibidi, Gräfelfing, Germany) and were incubated in standard conditions overnight. The next day, the inserts were removed to reveal a 500 µm cell-free gap. Then, the cells were treated with undiluted CFCS from *L. plantarum* L125 or *L. rhamnosus* GG. Untreated cells (control) were maintained in the cell culture medium, as mentioned above. Photographs were taken with a ZEISS Primovert light microscope (Zeiss, Göttingen, Germany) equipped with a digital camera (Axiocam ERc 5 s) at 0, 24 and 48 h post-treatment.

### 2.9. Statistical Analysis

Statistical differences in the in vitro experiments were analyzed using 2-tailed Student’s *t*-tests. A *p* < 0.05 was considered statistically significant. Results were expressed as the mean ± standard deviation of measurements. All experimental procedures were repeated three independent times unless otherwise stated.

## 3. Results

### 3.1. Genome Features

**Figure 1:**
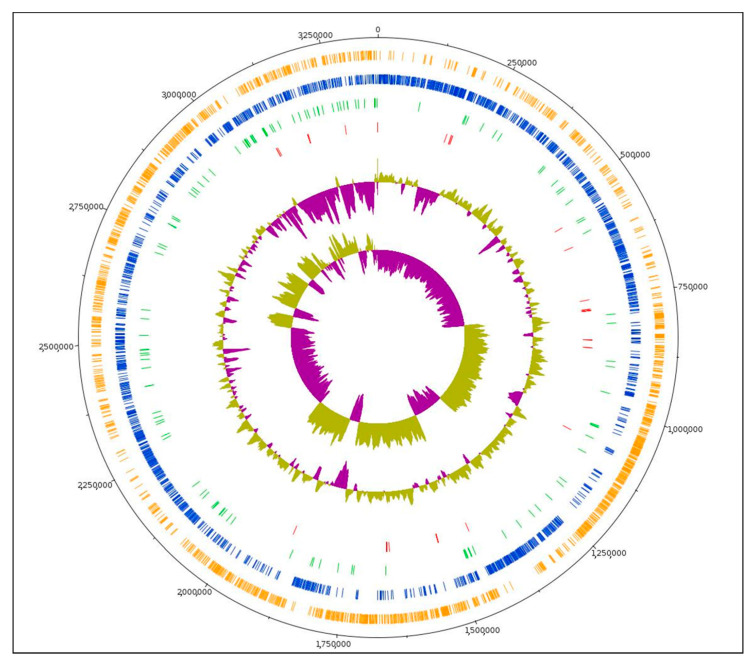
Circular genome map of *L. plantarum* L125. From the outer to inner circle, the information is displayed as follows: genome size (black), forward strand CDS (orange), reverse strand CDS (blue), pseudogenes (green), tRNA genes (red), GC content, GC skew.

The genomic characteristics of *L. plantarum* L125 were investigated using whole-genome sequencing and comprehensive bioinformatic analysis (Table 1), leading to the construction of its genome map (Figure 1). The complete genome of *L. plantarum* L125 consists of 3,354,135 bp with a GC content of 44.34%. The 3220 predicted genes include 3024 protein-coding sequences (CDSs), 126 pseudogenes, 62 tRNA genes, 4 rRNA genes as well as 4 ncRNAs. Both PGAP and CRISPRDetect (version 2.4) provided evidence that *L. plantarum* L125 does not carry CRISPR arrays. In addition, one intact prophage region with a length of 35 kb was identified (Table S2). Lastly, we did not identify any transferable genetic elements related to antibiotic resistance in the genome of *L. plantarum* L125, which agrees with previous in vitro findings [19].

### 3.2. Phylogenetic Analysis and Unique Genome Characteristics of L. plantarum L125

**Figure 2:**
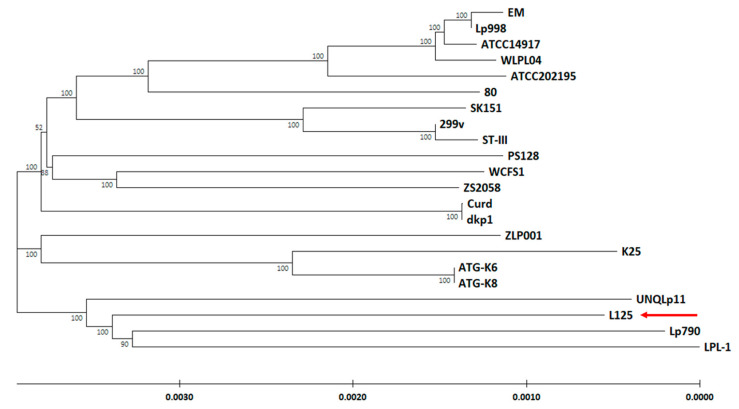
Neighbor-joining phylogenetic tree of *L. plantarum* L125 and 21 potential probiotic *L. plantarum* strains based on orthologous genes. The tree was constructed using 1000 bootstrap replicates, calculated by MEGAX (version 10.1.8). The red arrow indicates the position of *L. plantarum* L125 in the phylogenetic tree.

**Figure 3:**
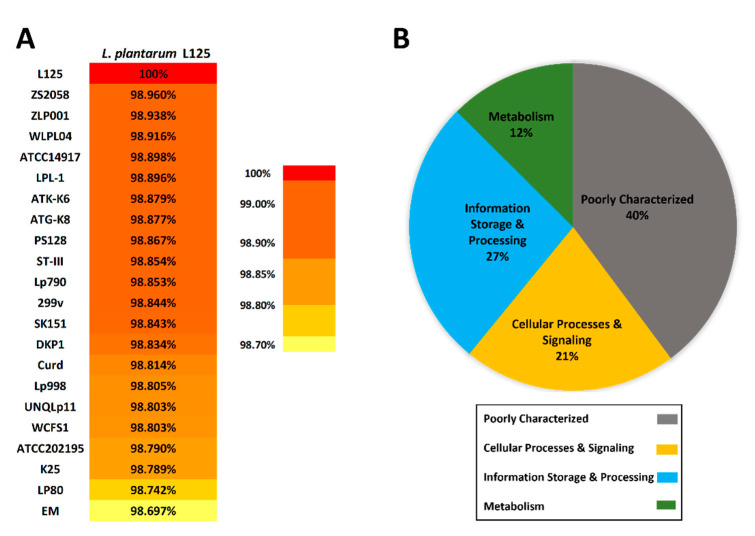
(**A**) Average Nucleotide Identity (ANI) matrix and similarity scores between the coding regions of *L. plantarum* L125 and the 21 *L. plantarum* strains. (**B**) *L. plantarum* L125 strain-specific genes, compared to the 21 *L. plantarum* strains, assigned to Clusters of Orthologous Groups (COGs) functional categories. “Function Unknown (S)” and “General Function Prediction only (R)” are depicted in the category termed “Poorly Characterized”.

*L. plantarum* L125 was classified as the species *Lactobacillus plantarum*, which is now known as *Lactiplantibacillus plantarum* [2,19]. Based on previous in vitro findings, *L. plantarum* L125 exhibits good probiotic potential [19,20]; therefore, in order to determine its phylogenetic position and relationship compared to other *L. plantarum* strains, we constructed a neighbor-joining phylogenetic tree, including 1000 bootstrap replicates (Figure 2). The phylogenetic tree is based on orthologous gene clusters and consists of *L. plantarum* L125 and 21 other potential probiotic *L. plantarum* strains. Among them, two well-established *L. plantarum* probiotic strains, *L. plantarum* WCFCS1 and *L. plantarum* 299v, were also included [6,42] (Figure 2). To assure the accurate phylogenetic placement of the newly sequenced strain, *Streptococcus pneumoniae* Hu17 and *Leuconostoc mesenteroides* SRCM102733 have been used as outgroups/controls (Figure S1). The reliability of the phylogenetic placement is also verified by ANI analysis, as *L. plantarum* L125 exhibited high ANI scores (>98.6%) with all *L. plantarum* strains (Figure 3A).

The vast majority of strains included in the tree have been isolated from fermented food products, mainly from kimchi, in countries located in East Asia, while only 2 strains were isolated from food products in European countries: *L. plantarum* L125 and *L. plantarum* Lp790. The abovementioned geographical correlation is reflected in the phylogenetic tree, as *L. plantarum* Lp790, which was isolated from Morlacco cheese in Italy and showed good probiotic potential in both in vitro and in vivo studies [43], is the closest evolutionary relative of *L. plantarum* L125 (Figure 2).

Furthermore, genome comparison of the 22 aforementioned *L. plantarum* strains revealed that *L. plantarum* L125 carries 220 unique genes. Strain-specific proteins were classified into COG functional categories. Notably, 60% of the genes code for proteins involved in fundamental cellular functions (Metabolism, Information Storage and Processing, Cellular Processes and Signaling). The remaining 40% of the genes are poorly characterized (Figure 3B). Overall, *L. plantarum* L125 appears to be part of the *L. plantarum* species and possesses a number of genes with important functions.

### 3.3. Functional Classification

**Figure 4:**
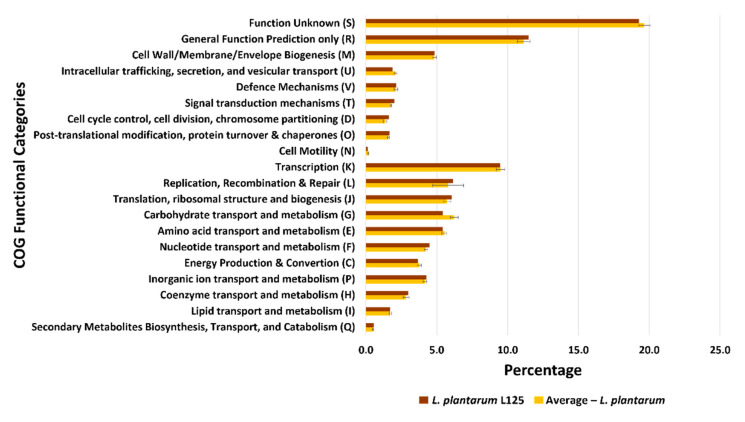
Comparison of the percentage of genes assigned to the COG functional categories of *L. plantarum* L125 (Brown bars) and of the 21 *L. plantarum* strains (Yellow bars). For each one of the 21 *L. plantarum* strains, the percentage of genes for each COG functional category was determined, and average values were calculated (Yellow bars). The values are depicted as mean ± standard deviation.

We conducted a comprehensive in silico analysis to describe the genomic traits of *L. plantarum* L125, as well as to compare them with the 21 selected *L. plantarum* strains. To gain a better insight into the functional characteristics of *L. plantarum* L125, its CDSs were allocated to COG and KEGG functional categories. The majority of the CDSs (94.48%) were assigned to 20 COG functional categories. Similarly, for the 21 *L. plantarum* strains, the CDSs of each strain were distributed into COG functional categories, and the average percentage for each COG category was calculated (Table S3). A comparison of the COG profile of *L. plantarum* L125 with the average values of the 21 *L. plantarum* strains revealed similar percentages in all COG functional categories (Figure 4). In both cases, the “Function Unknown (S)” was the most abundant category, followed by “General Function Prediction only (R)” and “Transcription (K)” (Figure 4). More precisely, *L. plantarum* L125 has 19.3% of its CDSs assigned to “Function Unknown (S)”, 11.5% to “General Function Prediction only (R)” and 9.5% to “Transcription (K)”.

**Figure 5:**
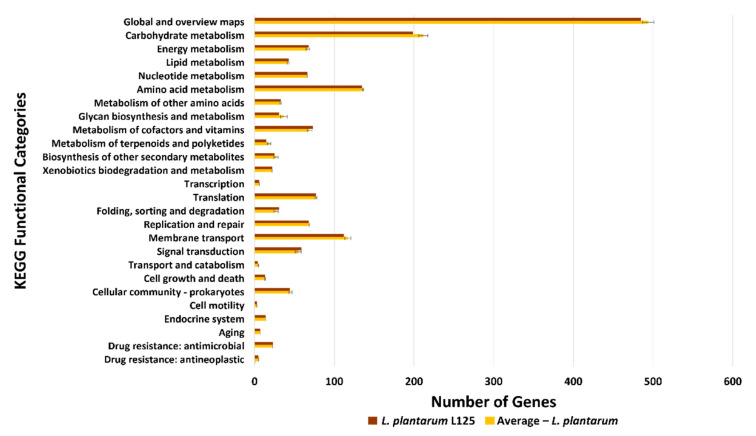
KEGG profiles comparison between *L. plantarum* L125 (Brown bars) and 21 *L. plantarum* strains (Yellow bars). For each one of the 21 *L. plantarum* strains, the number of genes in each KEGG functional category was determined, and average values were calculated (Yellow bars). The values are depicted as mean ± standard deviation.

Concomitantly, we performed KEGG analysis to uncover the variety and functionality of proteins coded by *L. plantarum* L125. More specifically, 53.20% of the *L. plantarum* L125 CDSs were classified into 39 KEGG functional categories and 180 pathways. These pathways notably include “biosynthesis of secondary metabolites” (ko: 01110; 170 genes), “microbial metabolism in diverse environments” (ko: 01120; 88 genes) and “ABC transporters” (ko: 02010; 78 genes). Regarding the capability of *L. plantarum* L125 to biosynthesize amino acids, KEGG pathway reconstruction showed that this strain can fully synthesize only 8 out of 20 amino acids: threonine, cysteine, methionine, lysine, histidine, arginine, proline and tryptophan (Table S4, Figures S2–S8), while it encodes part of the essential proteins involved in the biosynthesis of the other twelve amino acids. Furthermore, we observed that the KEGG profile of *L. plantarum* L125 is comparable to that of the other 21 *L. plantarum* strains included in the study (Figure 5, Table S5).

Moreover, we searched the genome of *L. plantarum* L125 for genes encoding enzymes involved in carbohydrate metabolism. We identified 76 genes that regulate the metabolism of a wide array of carbohydrates and assigned them into five CAZymes gene classes: 36 glycoside hydrolase (GH) genes, 31 glycosyltransferase (GT) genes, 5 carbohydrate-binding modules (CBMs) genes, 3 carbohydrate esterase (CE) genes and 1 Auxiliary Activity (AA) gene, (Table S6). Thus, *L. plantarum* L125 may be able to utilize several mono- and polysaccharides as energy sources and also synthesize complex molecules, such as chitin and cellulose. This finding could support the nomadic nature of the strain, common to *L. plantarum* strains [3].

### 3.4. Identification of Genes Implicated in Stress Response, Microbe–Host Interactions and Bacteriocin Biosynthesis

The genome of *L. plantarum* L125 was scoured for genetic loci implicated in the interaction with the host. In this context, genome annotation revealed that *L. plantarum* L125 possesses two genes coding for proteins mediating survival in the GI tract [44]: cation:proton antiporter and the PBP1A family penicillin-binding protein (Table 2). Furthermore, three genes involved in the acid tolerance mechanisms [45] of *L. plantarum* L125 were also identified: D-alanine--poly(phosphoribitol) ligase subunit (*dltA*), D-alanyl-lipoteichoic acid biosynthesis protein (*dltD*) and glutamate decarboxylase (*gadB*). Moreover, an F0F1-ATPase that consists of eight subunits, known for its role in acidic tolerance [46], and three bile salt hydrolases were also detected in the genome of *L. plantarum* L125. 

Exposure to extreme temperatures, prevalent in the food industry, can be stressful for bacteria and subsequently lead to the expression of heat and cold shock proteins. *L. plantarum* L125 carries five proteins involved in heat shock response (Table 2); molecular chaperone DnaJ, molecular chaperone DnaK, nucleotide exchange factor GrpE, chaperonin GroEL, co-chaperone GroES. Accordingly, survival in low temperatures can be mediated by three proteins of the cold shock protein family.

Moreover, *L. plantarum* L125 codes for a plethora of cell surface proteins (Table 2). More specifically, *L. plantarum* L125 contains six proteins carrying cell wall anchor domains (LPxTG motifs), as well as 1 gene encoding for a collagen-binding protein. Furthermore, three proteins with mucus-binding domains and two fibronectin-binding domain-containing proteins were also identified. Finally, the elongation factor Tu, a moonlighting protein with adhesin-like activity, was detected within the *L. plantarum* L125 genome.

**Figure 6:**
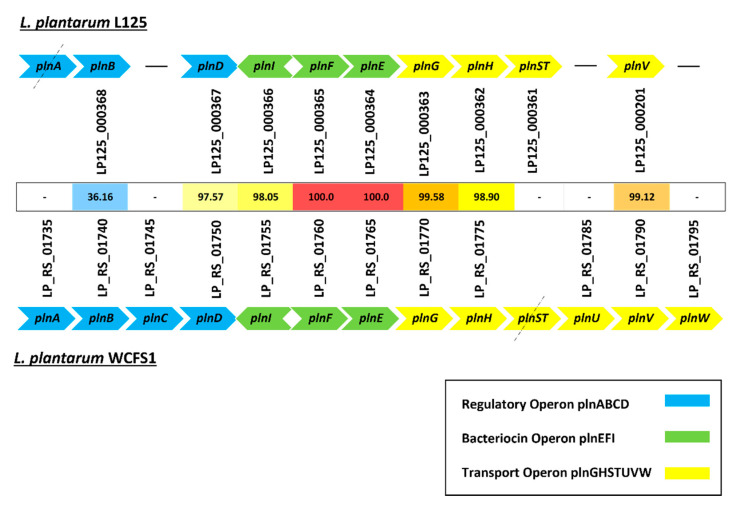
Detailed comparison of genes inside the pln locus (*plnABCD*, *plnEFI* and *plnGHSTUVW* operons) between *L. plantarum* L125 and *L. plantarum* WCFCS1. Black dashed lines represent pseudogenes, while black hyphens indicate gene absence. Protein identities are also indicated.

Over the last few years, genome analysis of numerous *L. plantarum* strains has revealed the presence of genetic loci responsible for the production of antimicrobial peptides, also known as bacteriocins [47]. To examine the capability of *L. plantarum* L125 to produce such antimicrobial peptides, we found that our strain possesses three genes that are crucial for the production of the class IIb bacteriocin: plantaricin EF. The abovementioned genes are homologous and exhibit high identity values to those of the probiotic strain *L. plantarum* WCFCS1 [48] (Figure 6). However, *L. plantarum* L125 lacks several genes of the *plnABCD* and *plnGHTUVW* operons, which are essential for transcriptional regulation and bacteriocin secretion, respectively [49] (Figure 6).

### 3.5. Investigation of Potential Health-Promoting Effects Induced by L. plantarum L125

**Figure 7:**
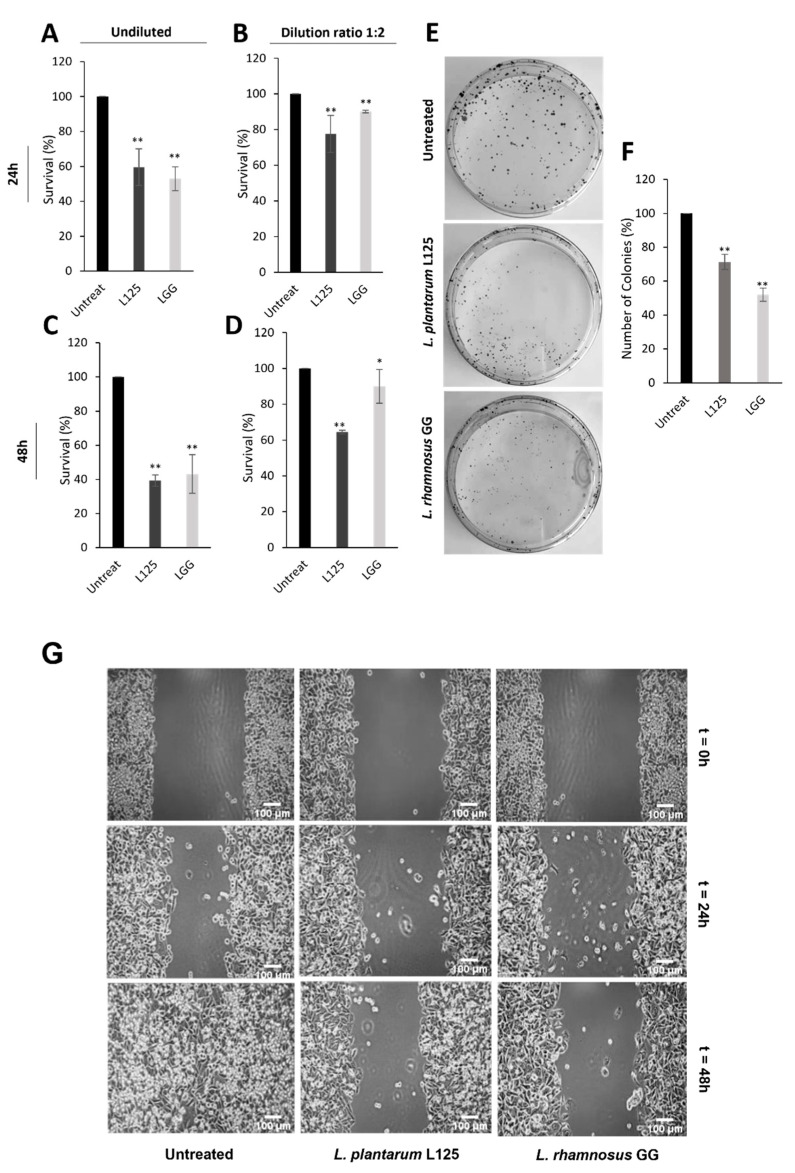
Time- and dose-dependent anti-proliferative, anti-clonogenic and anti-migration activity of *L. plantarum* L125-derived CFCS against the human adenocarcinoma cell line, HT-29. *L. rhamnosus* GG was used as a reference. The SRB colorimetric assay was used to evaluate the anti-proliferative activity of undiluted (**A**,**C**) or diluted at a ratio of 1:2 (**B**,**D**) *L. plantarum* L125 or *L. rhamnosus* GG-derived CFCS, after 24 (**A**,**B**) or 48 (**C**,**D**) hour treatments. (**E**) Representative photos of the colony formation assay results, showing the anti-clonogenic potential of undiluted *L. plantarum* L125 and *L. rhamnosus* GG CFCS after 48 h treatments. (**F**) Quantitative results of the colony formation assay for the reference and tested strain. (**G**) The anti-migration capacity of *L. plantarum* L125- or *L. rhamnosus* GG-derived CFCS, evaluated by the wound healing assay. Photos were taken at 0, 24 and 48 h post-incubation with undiluted CFCS. Scale bar, 100 μm. Data are presented as the mean ± standard deviation. * *p* < 0.05, ** *p* < 0.001 compared to control, untreated cells.

Potential probiotic strains can induce a variety of beneficial actions when interacting with the host. In this study, we explored the anti-proliferative activity of *L. plantarum* L125 CFCS against the human colon adenocarcinoma cell line, HT-29. For that reason, cells were treated with undiluted or diluted at a ratio of 1:2 CFCS and cell survival was estimated using the SRB colorimetric assay. *L. rhamnosus* GG was used as a reference strain due to its well-characterized cytotoxic and anti-proliferative properties [50]. CFCS treatments induced a significant time- and dose-dependent effect (Figure 7A–D, *p* < 0.01). More specifically, the undiluted *L. plantarum* L125 CFCS decreased cell survival by 40 and 60% after 24 and 48 h treatments, respectively (Figure 7A,C). The reference strain induced similar effects. Furthermore, we sought to determine the anti-clonogenic potential of *L. plantarum* L125-derived CFCS by employing the colony formation assay. Indeed, the undiluted CFCS significantly reduced the number of viable colonies compared to control, untreated cells (*p* < 0.01) (Figure 7E,F). Finally, the anti-migration capacity of the undiluted CFCS was assessed by the wound healing assay. Notably, HT-29 co-incubation with CFCS limited cell migration (Figure 7G). On the other hand, wound healing of the untreated sample was completed after 48 h (Figure 7G).

## 4. Discussion

In this study, we announce the draft genome sequence of *L. plantarum* L125, a strain presenting biotechnological interest, that was originally isolated from a fermented sausage [19]. The complete genome of the strain consists of 3,354,135 bp with a GC content of 44,34%, and it contains one prophage region and no CRISPR arrays. *L. plantarum* L125, although originally isolated from meat products, may be able to adapt to a variety of niches, as suggested by the fact that 88 of its genes are assigned to the KEGG pathway “microbial metabolism in diverse environments”. Indeed, *L. plantarum* strains are able to colonize a wide range of habitats such as the human GI tract, meat, fish, vegetables, dairy and other fermented products [1]. The nomadic lifestyle of the species is mirrored in the vast genetic diversity that *L. plantarum* strains present [51]. Previous reports indicate that during the environmental adaptation process, genomic changes may occur [52,53]; however, a strong link between genome content and niche adaptation of *L. plantarum* has not been established yet [3]. Indeed, our phylogenetic analysis did not reveal any grouping of the studied *L. plantarum* strains based on their isolation source. Interestingly, we observed that the closest evolutionary relative of *L. plantarum* L125 is *L. plantarum* Lp790, the only other strain that was isolated in Europe and, more specifically, from Italian dairy products [43]. The abovementioned genome alterations during niche adaptation, including gene gain/loss events, may affect genetic clusters associated with amino acid biosynthesis [54]. In this study, we showed that *L. plantarum* L125 possesses complete biosynthetic pathways for eight out of the 20 amino acids (Figures S2–S8), underlining the need for amino acid supply from nutrient-dense environments. Likewise, the KEGG reconstruction pathway revealed that all studied *L. plantarum* strains exhibit identical capability regarding amino acid biosynthesis.

A prerequisite for microbe–host interactions to occur is the tolerance of the host niche. A previous report revealed the ability of *L. plantarum* L125 to survive in highly acidic and bile-rich environments [19]. In fact, this strain did manage to withstand the abovementioned stress conditions, which are similar to those prevailing in the human GI tract [19]. In this study, we found numerous proteins that support the previous in vitro findings and are associated with either acid tolerance or bile salt resistance (Table 2). Furthermore, according to the same study, *L. plantarum* L125 tolerance to bile salts is accompanied by bile salt hydrolase activity. Indeed, a comprehensive bioinformatical analysis revealed the presence of bile salt hydrolases within the *L. plantarum* L125 genome (Table 2). Moreover, probiotics intended for biotechnological application should tolerate heat or/and cold stress conditions [55], and therefore, the presence of heat and cold shock proteins within their genome is regarded as a desirable trait. In this context, *L. plantarum* L125 was detected in high counts in Greek traditional dry fermented sausages that were stored at 4 °C for 160 days [20]. These findings indicate that the cold-shock family proteins we identified (Table 2) are functional and correlated with their viability at low temperatures.

The ability of lactobacilli to adhere to and interact with intestinal surfaces is considered to be crucial for their probiotic action [56]. A number of cell surface molecules such as polysaccharides and proteins have been associated with this phenotype [57]. More specifically, probiotic bacteria utilize collagen-, mucin- and fibronectin-binding proteins, as well as LPXTG domain-containing proteins, to attach to the host intestinal epithelial cells or mucosa [58]. In addition, the adhesion capability of lactobacilli is also supported by several moonlighting proteins, such as EF-Tu [59], which, among other functions, can exhibit adhesin-like activity [56]. In this study, we identified numerous cell surface adhesins and moonlighting proteins in *L. plantarum* L125 (Table 2). Future studies will explore the adhesion capacity of the strain in vitro and will focus on the specific mechanisms mediating this effect, as well as the biological significance of this interaction for the host cell.

Concerning the antimicrobial activity of probiotic strains, they can exert inhibitory effects by utilizing a great variety of mechanisms. Indeed, direct antimicrobial activity of *L. plantarum* can be induced by the secretion of inhibitory compounds, such as bacteriocins [60], fatty acids, ethanol and hydrogen peroxide [61], or by competitive pathogen exclusion [62]. In this vein, the bacteriocin produced by *L. plantarum* ATCC 8014 limited the proliferation of *Staphylococcus aureus* 99308 in a mouse model of mandibular fracture postoperative infection [63]. Furthermore, some probiotic strains can stimulate the immune response of the host, leading to pathogen clearance [64]. Our results corroborate previous studies showing that *L. plantarum* L125 does not present bacteriocin-like activity [19], although it carries three genes encoding for plantaricin EF (Figure 6). In greater detail, it lacks essential genes for transcriptional regulation and secretion, alluding to the fact that the plantaricin cluster may not be functional. The ability of *L. plantarum* L125 to inhibit pathogen colonization, expansion and biofilm formation by mechanisms other than bacteriocin synthesis will be studied in the future.

Another significant health-promoting property that specific probiotic strains possess is the ability to regulate cell cycle progression and cell death [64]. Indeed, previous studies from our lab have shown that potential probiotics, such as *L. pentosus* B281 and *L. paracasei* K5, can induce cell death in species- and strain-specific fashion [40,65]. In this context, the administration of viable *L. casei* ATCC 393 cells to a mouse model bearing CT26 tumors led to a reduction in tumor volume via the induction of apoptotic cell death [66]. Similarly, ferrichrome, isolated from *L. casei* ATCC334 CFCS, exerted tumor-suppressive effects in a BALB/c xenograft model that were also attributed to the induction of apoptosis and, more specifically, to the c-Jun N-terminal kinase (JNK) signaling pathway {15]. However, potential probiotic strains can also induce cytotoxic effects by alternative mechanisms, such as immunogenic cell death [67]. For example, the oral administration of heat-killed *L. plantarum* BF-LP284 to a murine syngeneic model of sarcoma and resulted in the inhibition of tumor growth and the stimulation of anti-tumor immune responses [68]. In the present study, we observed that CFCS of *L. plantarum* L125 can effectively limit the proliferation and migration capacity of HT-29 cells. HT-29 cells were selected in this study as an in vitro model of the human colon. The observed effects were mediated in a time- and dose-dependent manner and were comparable to the activity of *L. rhamnosus* GG, a well-studied probiotic strain (Figure 7). Of note, the observed reduction in cell viability was not due to the acidic pH of CFCS (data not shown). On the other hand, cell surface molecules and/or excreted metabolites may mediate these anti-proliferative actions. Regarding the nature of these active compounds, exopolysaccharides (EPS), peptidoglycans and conjugated linolenic acids (CLA), as well as S-layer proteins, have been implicated in the induction of cell death [69,70]. Interestingly, we have located clusters for EPS and CLA biosynthesis in the genome of *L. plantarum* L125 (data not shown). The latter was almost identical to the functional CLA biosynthesis cluster found in *L. plantarum* ZS2058 [71]. However, further studies are needed to determine their functionality and the potential contribution of these molecules to the observed anti-proliferative effects.

## 5. Conclusions

In this study, we presented the whole genome sequence of *L. plantarum* L125 and performed comprehensive bioinformatic analysis to locate genes involved in the probiotic phenotype. We found the strain codes for proteins supporting survival and adaptation in the gastrointestinal niche, as well as tolerance to conditions prevalent in the food industry. Concerning the potential health benefit of the strain, we observed that the CFCS from *L. plantarum* L125 can induce anti-proliferative, anti-clonogenic and anti-migration effects on the colon adenocarcinoma cell line, HT-29. Additional studies are needed to validate the putative anticancer potential of the strain in animal models of tumorigenesis and in the clinical setting. Subsequently, its incorporation in the functional food industry will further be examined.

## Figures and Tables

**Table 1:** *L. plantarum* L125 genome features.

Attribute	Values
Genome Size (bp)	3,354,135
GC content (%)	44.34
Total Genes	3220
CDS (protein)	3024
Pseudogenes	126
tRNA genes	62
rRNA genes	4
ncRNA genes	4

**Table 2:** List of proteins encoded by *L. plantarum* L125, involved in stress response and host–microbe interactions.

Locus Tag	Description	Role
LP125_003204	cation:proton antiporter	GI tract survival
LP125_001869	PBP1A family penicillin-binding protein	GI tract survival
LP125_002196	D-alanine--poly(phosphoribitol) ligase subunit DltA	Acid tolerance
LP125_002199	D-alanyl-lipoteichoic acid biosynthesis protein DltD	Acid tolerance
LP125_001705	glutamate decarboxylase	Acid tolerance
LP125_000817	F0F1 ATP synthase subunit epsilon	Acid tolerance
LP125_000818	F0F1 ATP synthase subunit beta	Acid tolerance
LP125_000819	F0F1 ATP synthase subunit gamma	Acid tolerance
LP125_000820	F0F1 ATP synthase subunit alpha	Acid tolerance
LP125_000821	F0F1 ATP synthase subunit delta	Acid tolerance
LP125_000822	F0F1 ATP synthase subunit B	Acid tolerance
LP125_000823	F0F1 ATP synthase subunit C	Acid tolerance
LP125_000824	F0F1 ATP synthase subunit A	Acid tolerance
LP125_003090	choloylglycine hydrolase family protein	Bile Resistance
LP125_000497	choloylglycine hydrolase family protein	Bile Resistance
LP125_000993	linear amide C-N hydrolase	Bile Resistance
LP125_001391	LPXTG cell wall anchor domain-containing protein	Cell surface protein
LP125_001882	LPXTG cell wall anchor domain-containing protein	Cell surface protein
LP125_001897	LPXTG cell wall anchor domain-containing protein	Cell surface protein
LP125_003116	LPXTG cell wall anchor domain-containing protein	Cell surface protein
LP125_000218	LPXTG cell wall anchor domain-containing protein	Cell surface protein
LP125_001232	LPXTG cell wall anchor domain-containing protein	Cell surface protein
LP125_000997	collagen binding protein	Adhesion
LP125_002620	MucBP domain-containing protein	Adhesion
LP125_000275	MucBP domain-containing protein	Adhesion
LP125_000616	MucBP domain-containing protein	Adhesion
LP125_002390	NFACT family protein	Adhesion
LP125_000010	NFACT family protein	Adhesion
LP125_002930	elongation factor tu	Adhesion
LP125_002193	molecular chaperone DnaJ	Heat Stress
LP125_002192	molecular chaperone DnaK	Heat Stress
LP125_002191	nucleotide exchange factor GrpE	Heat Stress
LP125_001567	chaperonin GroEL	Heat Stress
LP125_001568	co-chaperone GroES	Heat Stress
LP125_002661	cold-shock protein	Cold Stress
LP125_002795	cold-shock protein	Cold Stress
LP125_003063	cold-shock protein	Cold Stress

## Data Availability

The *L. plantarum* strain L125 genome sequence has been deposited at DDBJ/ENA/GenBank under the accession JAIGOE000000000. The version described in this paper is version JAIGOE010000000.

## Supplementary Materials

The following are available online at https://www.mdpi.com/article/10.3390/biomedicines9111718/s1, Figure S1: Neighbor-joining phylogenetic tree based on orthologous genes, of *L. plantarum* L125 and 21 *L. plantarum* strains. Figure S2: The KEGG pathway of Glycine, Serine and Threonine Metabolism (ko: 00260). Figure S3: The KEGG pathway of Cysteine and Methionine Metabolism (ko: 00270). Figure S4: The KEGG pathway of Lysine Metabolism (ko: 00300). Figure S5: The KEGG pathway of Arginine Metabolism (ko: 00220). Figure S6: The KEGG pathway of Arginine and Proline Metabolism (ko: 00330). Figure S7: The KEGG pathway of Histidine Metabolism (ko: 00340). Figure S8: The KEGG pathway of Tryptophan Metabolism KEGG pathway (ko: 00400). Table S1: The probiotic properties of the 21 *L. plantarum* strains, used in the comparative genomic analysis. Table S2: Prophage sequences in genome assembly of *L. plantarum* L125 by PHAge Search Tool Enhanced Release (PHASTER). Table S3: Detailed presentation of the percentages of genes assigned to the COG functional categories of *L. plantarum* L125 and of the 21 *L. plantarum* strains. Table S4: List of complete KEGG modules and pathways that reflect the amino acid biosynthesis capability of *L. plantarum* 125. Table S5: Detailed presentation of the number of genes assigned to the KEGG functional categories of *L. plantarum* L125 and of the 21 *L. plantarum* strains. Table S6: *L. plantarum* L125 genes assignment into CAZymes families aided by the CAZy database.
