# Supplementary material for: Genomic and Phylogenetic Analysis of Lactiplantibacillus plantarum L125, and Evaluation of Its Anti-Proliferative and Cytotoxic Activity in Cancer Cells
Source: Biomedicines. 2021 Nov 19;9(11):1718. doi: 10.3390/biomedicines9111718 (PMC8615743; doi:10.3390/biomedicines9111718)
Supplement: Supplementary file 1 [file biomedicines-09-01718-s001.zip › Tegopoulos et al. Supplementary Figures.pdf]

Supplementary Materials

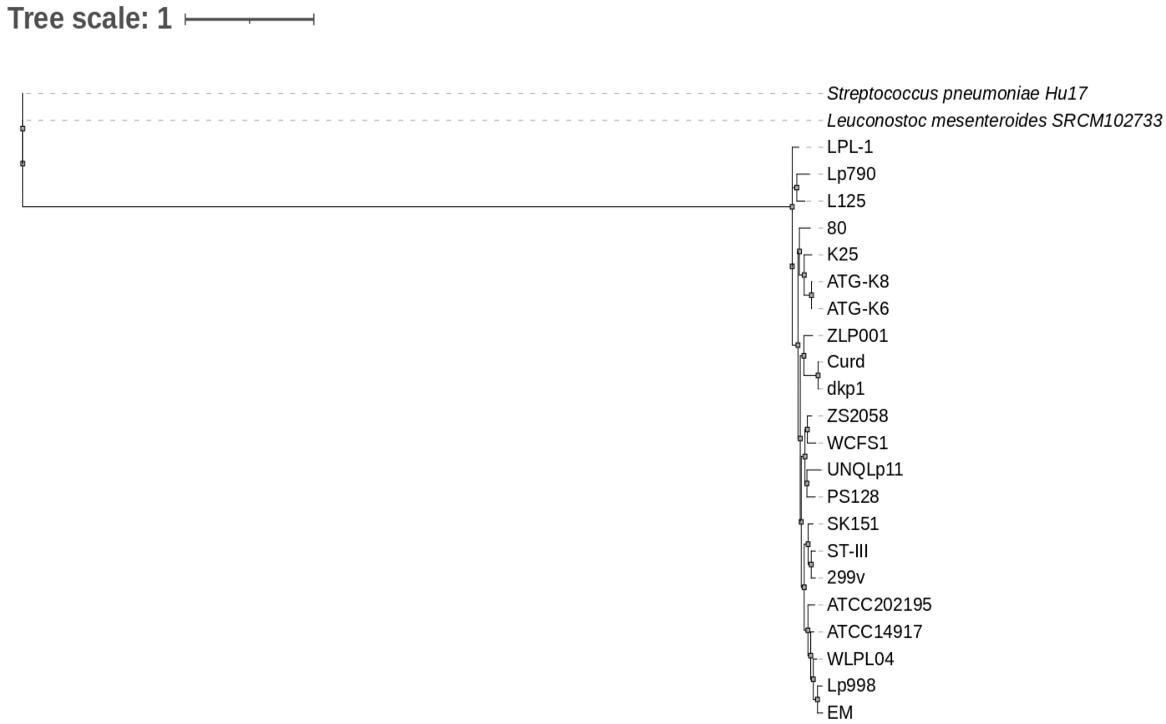

Figure S1. Neighbor-joining phylogenetic tree based on orthologous genes, of *L. plantarum* L125 and 21 *L. plantarum* strains. *Streptococcus pneumoniae* Hu17 and *Leuconostoc mesenteroides* SRCM102733 have been used as outgroups/controls.

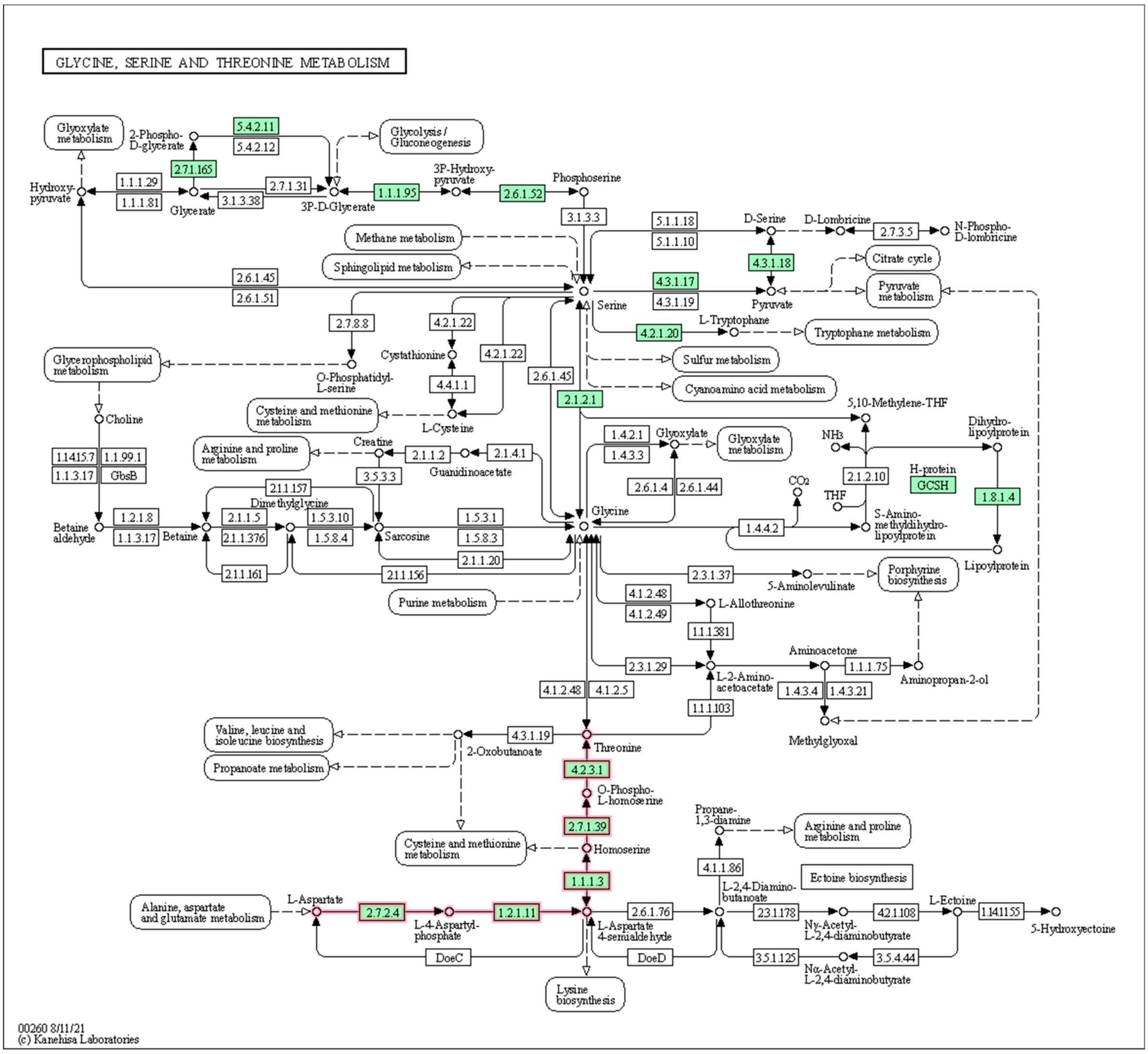

Figure S2: The KEGG pathway of Glycine, Serine and Threonine Metabolism (ko: 00260). Every box represents a protein involved in the pathway. *L. plantarum* L125 possesses the proteins presented in green colored boxes. Green boxes outlined with pink color make up the complete threonine biosynthesis module (M00018), indicating the ability of *L. plantarum* L125 to synthesize Threonine.

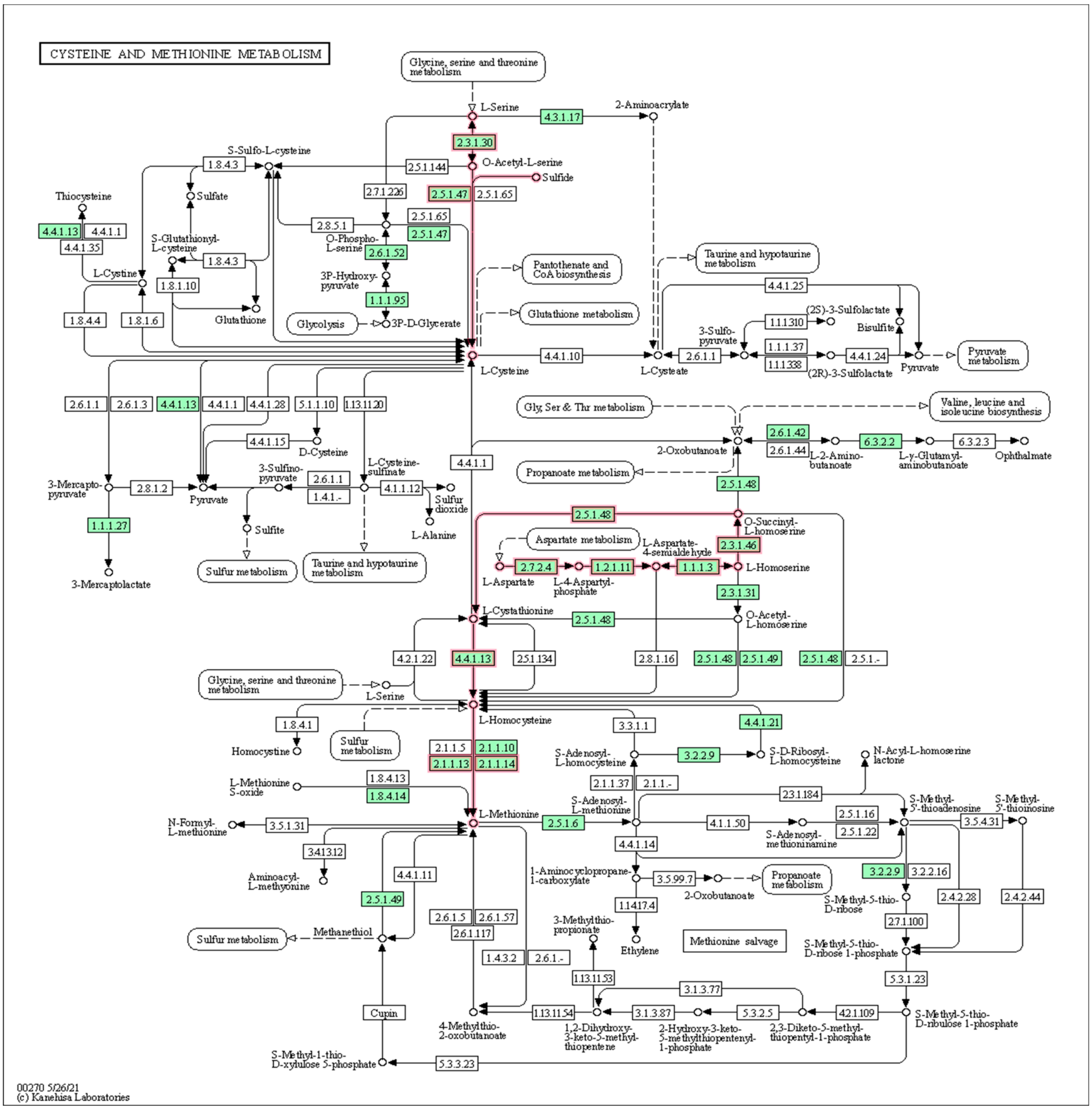

Figure S3. The KEGG pathway of Cysteine and Methionine Metabolism (ko: 00270). Every box represents a protein involved in the pathway. *L. plantarum* L125 possesses all the proteins presented in green colored boxes. Green boxes outlined with pink color make up the complete cysteine biosynthesis module (M00021) as well as the complete methionine biosynthesis module (M00017), indicating the ability of *L. plantarum* L125 to synthesize Cysteine and Methionine.

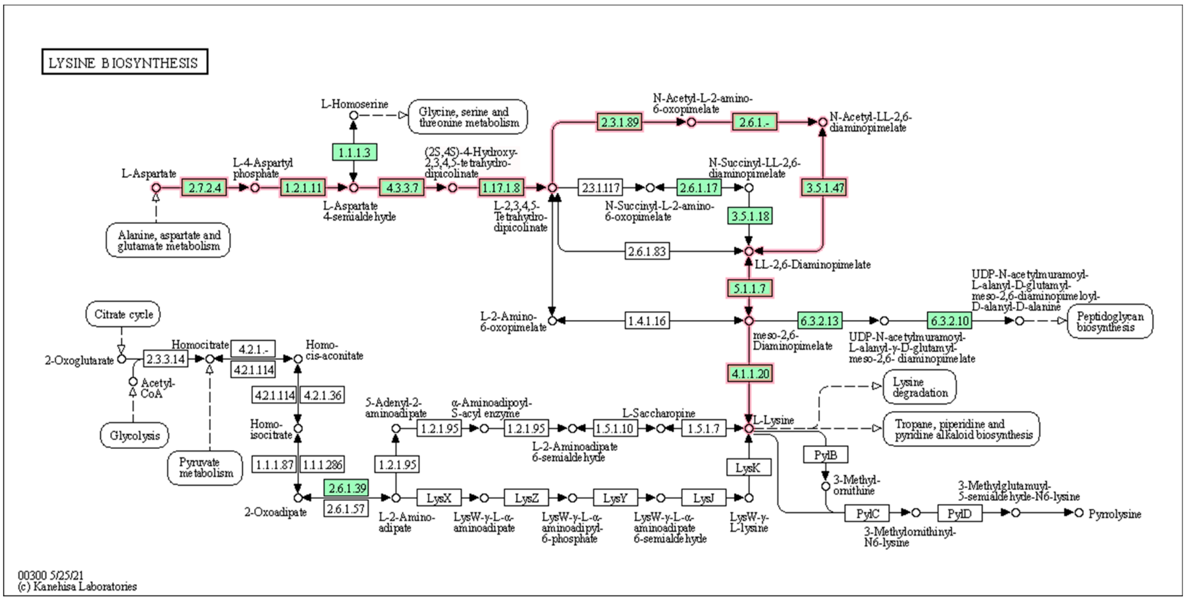

Figure S4. The KEGG pathway of Lysine Metabolism (ko: 00300). Every box represents a protein involved in the pathway. *L. plantarum* L125 possesses all the proteins presented in green colored boxes. Green boxes outlined with pink color make up the complete lysine biosynthesis module (M00525), indicating the ability of *L. plantarum* L125 to synthesize Lysine.

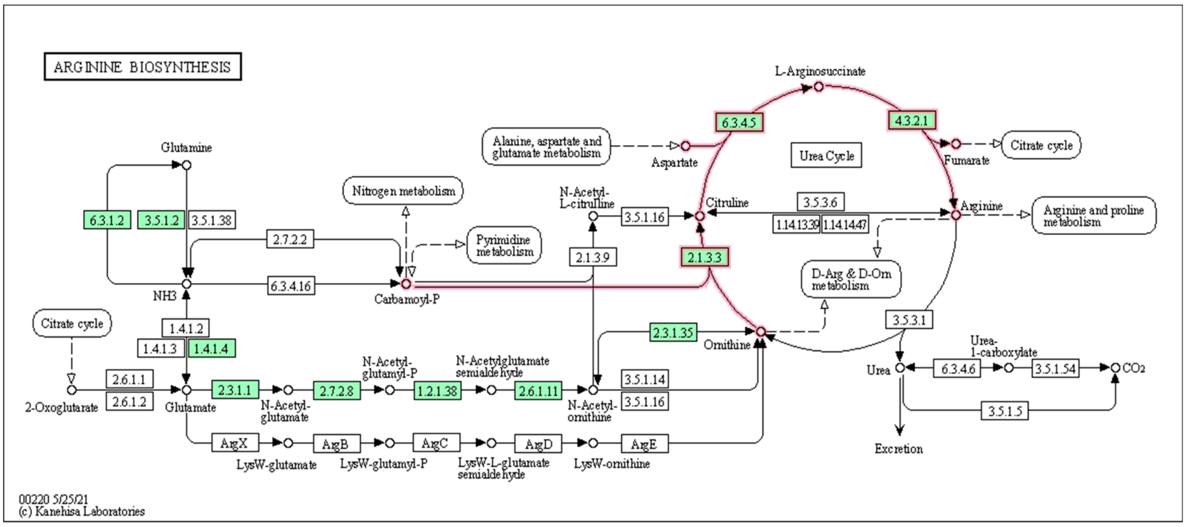

Figure S5. The KEGG pathway of Arginine Metabolism (ko: 00220). Every box represents a protein involved in the pathway. *L. plantarum* L125 possesses all the proteins presented in green colored boxes. Green boxes outlined with pink color make up the complete arginine biosynthesis module (M00844), indicating the ability of *L. plantarum* L125 to synthesize Arginine.

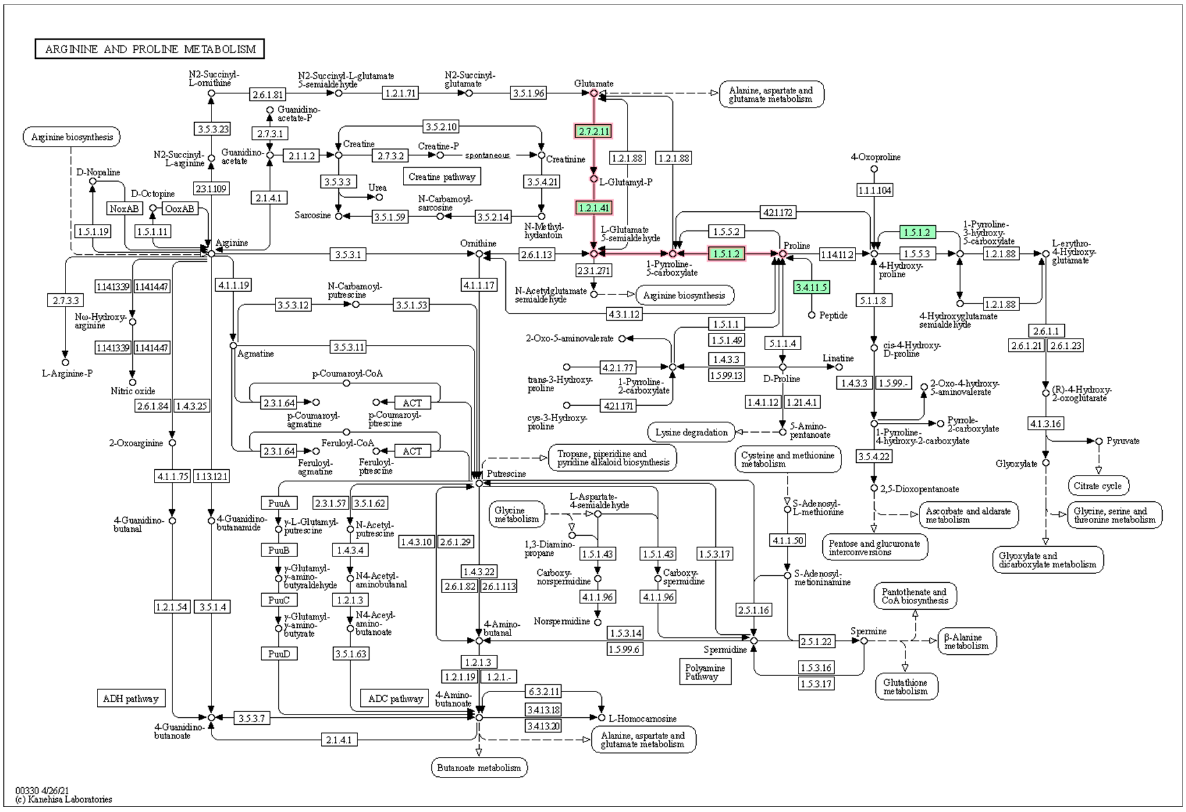

Figure S6. The KEGG pathway of Arginine and Proline Metabolism (ko: 00330). Every box represents a protein involved in the pathway. L. plantarum L125 possesses all the proteins presented in green colored boxes. Green boxes outlined with pink color make up the complete proline biosynthesis module (M00015), indicating the ability of L. plantarum L125 to synthesize Proline.

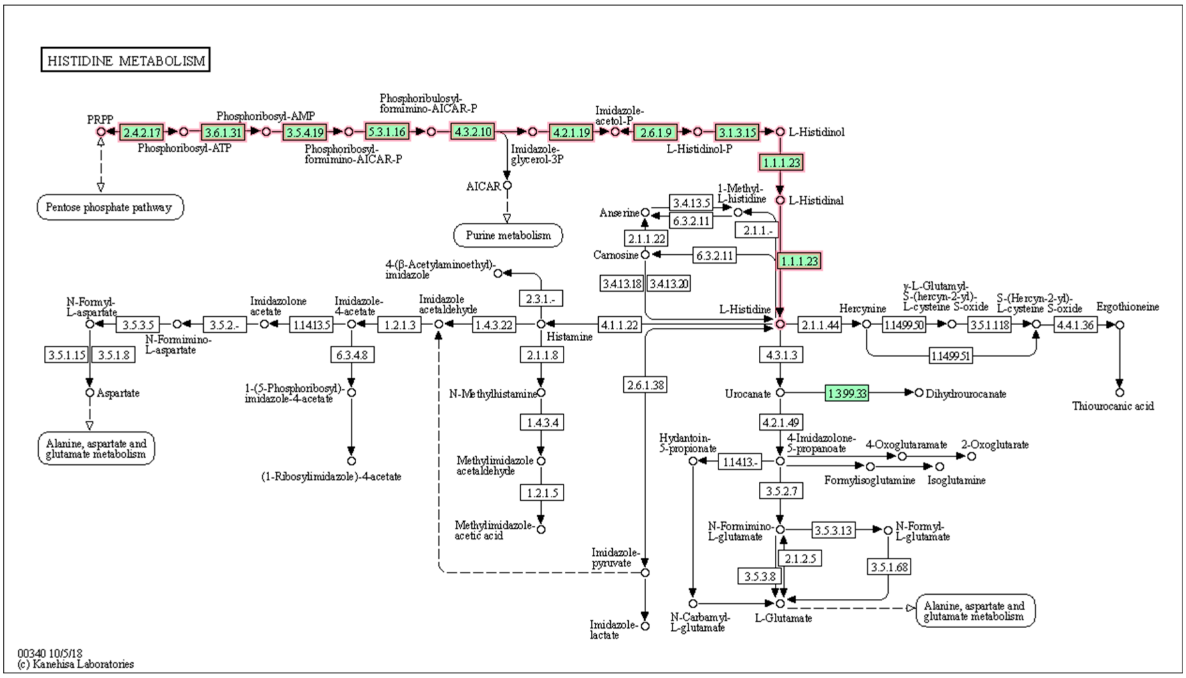

Figure S7. The KEGG pathway of Histidine Metabolism (ko: 00340). Every box represents a protein involved in the pathway. *L. plantarum* L125 possesses all the proteins presented in green colored boxes. Green boxes outlined with pink color make up the complete histidine biosynthesis module (M00026), indicating the ability of *L. plantarum* L125 to synthesize Histidine.

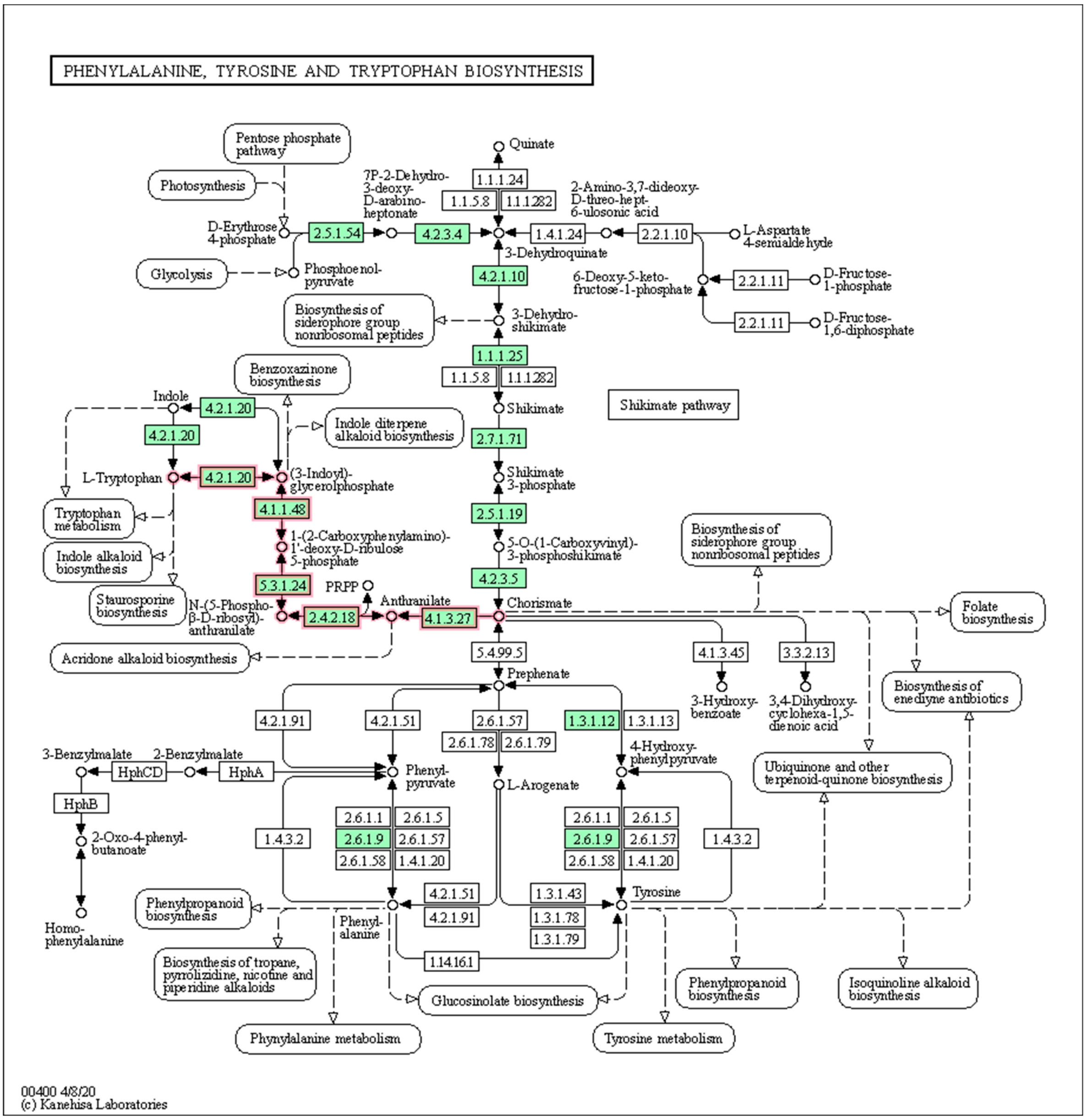

Figure S8. The KEGG pathway of Tryptophan Metabolism KEGG pathway (ko: 00400). Every box represents a protein involved in the pathway. *L. plantarum* L125 possesses all the proteins presented in green colored boxes. Green boxes outlined with pink color make up the complete tryptophan biosynthesis module (M00023), indicating the ability of *L. plantarum* L125 to synthesize Tryptophan.
